# *MiR-199a/b-3p* inhibits colorectal cancer cell proliferation, migration and invasion through targeting *PAK4* and *BCAR3*

**DOI:** 10.1186/s40001-022-00750-8

**Published:** 2022-07-16

**Authors:** Junjie Hou, Xuguang Mi, Ning Liu, Xiaonan Li, Xiao-nan Li, Ying Yang, Xiaodan Lu, Yanqiu Fang, Ning-Yi Jin

**Affiliations:** 1 Medical College, Yanbian University, Yanji, 133002 China; 2 Tumor Biotherapy Center, Jilin Province People’s Hospital, Changchun, China; 3 General Surgery of The First Clinical Hospital of Jilin Academy of Chinese Medicine Sciences, Changchun, China; 4 Changchun Institute of Veterinary Medicine, Chinese Academy of Agricultural Sciences, Changchun, China

**Keywords:** miR-199a/b-3p, Viability, Mobility, *PAK4*, *BCAR3*, Colorectal cancer (CRC)

## Abstract

**Background:**

Colorectal cancer (CRC) is one of the leading causes of cancer-related death worldwide. P21 activated kinase 4 (*PAK4*) and Breast cancer anti-estrogen resistance 3 (*BCAR3*) have been reported to be involved in numerous aspects in tumorous progression. In this study, we propose to screen multi-targeted microRNAs. (miRNAs), which simultaneously inhibit neoplastic evolution through suppressing the transcription of target genes.

**Methods:**

MTT and Colony formation assays measured cell’s viability and proliferation. Scratch wound and Transwell assays detected the ability in migration and invasion for SW116 cells. The multi-targeted microRNAs of *PAK4* and *BCAR3* were predicted using bioinformatics analysis and verified by conducting dual luciferase reporter assay, western blot and qRT-PCR that could detect the expression levels of *miR-199a/b-3p*.

**Results:**

The knockdown of *PAK4* significantly impeded proliferation and colony formation of SW1116 cells when the knockdown of *BCAR3* hindered migration and invasion of SW1116 cells. MiR-199a/b-3p directly targeted the 3'-UTR of *PAK4* and *BCAR3*, further effected proliferation, colony formation, migration, and invasion of SW1116 cells. *PAK4* or *BCAR3* overexpression could partially reversed inhibitory effects of miR-199a/b-3p.

**Conclusions:**

These results provided a new multi-targeted cite for cancerous suppressant to improve the prognosis of CRC inpatients.

## Introduction

Colorectal cancer (CRC) is the fourth most deadly cancer in the world, accounting for 10 percent of diagnosed cancers and cancer-related deaths worldwide [1]. Nearly 900,000 people die of CRC each year [2]. Owing to aging population and dietary habits, the risk of CRC is high in high-income countries. Some other factors such as obesity, smoking, and lack of physical activity also increase the risk of CRC [3]. Much progress has been made in the treatment of CRC with surgical resection, adjuvant chemotherapy and radiotherapy. However, the recurrence and metastasis rate of CRC are still high [4]. Therefore, it is essential to elucidate the underlying mechanisms involving in the progression and development of this disease.

P21 activated kinase 4 (*PAK4*) is a member of Group 2 of the p21 activated kinases, which are downstream effectors of CdC42 and Rac1 and participate in biological behaviors of cancer [5]. It has been shown to suppress cell proliferation and its expression was upregulated in various cancers [6, 7]. A study on glioblastoma suggested that *PAK4* was down-regulated by miR-485 and inhibited the malignant biological behavior of glioblastoma cells [8]. In esophageal cancer, miR-199a-3p was reported to reduce cell proliferation by targeting *PAK4* [9]. Moreover, some studies have shown that certain miRNAs had suppressive effects on CRC cells through targeting *PAK4* [10, 11]. The breast cancer anti-estrogen resistance 3 (*BCAR3*) is the member of novel Src homology 2 (SH2) containing protein (NSP) family [12]. Recent study showed that overexpression of *BCAR3* regulated cytoskeletal rearrangement [13]. It was also reported that *BCAR3* overexpression increased cell migration and invasion in endometriosis [14]. However, the role of *BCAR3* in CRC remains unclear.

In recent years, miRNAs have been shown to play a significant role in cancer occurrence, metastasis, and progression [15]. They are small noncoding RNAs of about 22 nucleotides in length and are crucial post-transcriptional and epigenetic regulators of gene expression in eukaryotes [16]. Increasing research have indicated that some miRNAs are important modulators on the progression of CRC [17, 18].

In this study, we aimed to explore the role of *PAK4* and *BCAR3* in CRC. We silenced the expression of *PAK4* and *BCAR3* separately and found that these two genes had different role on CRC cells. In view of the diversity of miRNA targets, we screened out miRNAs that inhibited both genes through the bioinformatics database. Surprisingly, we identified miR-199a/b-3p as the potential target miRNA of *PAK4* and *BCAR3*. Our results suggested that miR-199a/b-3p inhibited the proliferation, colony formation of CRC cells through targeting *PAK4*. Meanwhile, the inhibitory effects on migration and invasion were regulated by targeting *BCAR3*.

## Materials and methods

### Cell line and cell culture

Human CRC cells (SW1116) were obtained from the Biochemistry and Cell Biology at the Chinese Academy of Sciences (Shanghai, China). SW1116 cells were cultured in RPMI-1640 medium with 10% fetal bovine serum (FBS) and 1% penicillin/streptomycin. All cells were cultured in humidified incubator at 37 °C with 5% CO2.

### Cell viability by MTT assay

3-(4,5-Dimethylthiazol-2-yl)-2,5-diphenyltetrazolium bromide (MTT) was purchased from Sigma-Aldrich. SW1116 cells were seeded at 10,000 cells per well in 96-well plates. Cell proliferation was determined at 24, 48, and 72 h using MTT assay. MTT was dissolved in phosphate buffered saline solution at the concentration of 5 mg/ml. After incubated with 20 μl of MTT for 4 h at 37 °C, the medium was aspirated and dimethyl sulfoxide (DMSO) was added to dissolve formazan. The absorbance was measured at a wavelength of 570 nm using an automated Microplate Reader. The experiment was performed in triplicate.

### Colony formation assay

After the treatments of SW1116 cells, cells were trypsinized to single-cell suspensions of 1000 cells and plated in 6-well plates. The plates were incubated at 37 °C in 5% CO_2_until sufficiently large clones were formed. The colonies were fixed with4% paraformaldehyde and stained with 0.5% crystal violet for 30 min.

### Transfection assay

SW1116 cells were seeded into 6-well plates the day before transfection. The transfection was performed using Lipofectamine 3000 (Invitrogen) according to the manufacturer’s instructions.

### Target prediction

Target miRNAs for *PAK4* and *BCAR3* were predicted using the TargetScan database (http://www.targetscan.org) and DIANA database (http://diana.imis.athena-innovation.gr).

### RNA extraction and real-time PCR

Total RNA from cells was extracted using Transzol reagent (Transgen) and then was converted into cDNA using the Transcript miRNA First-Strand cDNA Synthesis SuperMix (Transgen). In the 7500 Real-Time PCR System (ABI), quantitative reverse transcription was carried out in a 20 ul reaction system. qRT-PCR primers for *PAK4* were F: 5′-TCCCCCTGAGCCATTGTG-3′, R: 5′-TGACCTGTCTCCCCATCCA-3′.qRT-PCR primers for *BCAR3* were F: 5′-GCGGTGGAACTGAAGGATTC-3′, R: 5′ -TGGCAGTTTGGGTGTACTGG-3′. GAPDH was used as internal control: 5′-GGACCTGACCTGCCGTCTAG-3′ and 5′-GTAGCCCAGGATGCCCTTGA-3′. qRT-PCR primers for miR-199a/b-3p was F: 5′-GTCACAGTAGTCTGCACAT-3′. U6 was used as internal control: 5′-CTCGCTTCGGCAGCACA-3′ and 5′-AACGCTTCACGAATTTGCGT-3′. The 2^−ΔΔCt^ method was used to analyze the expression levels of gene and miRNA.

### Dual luciferase reporter assay

The total cDNA from the SW1116 cells was used to amplify the 3′-UTR of *PAK4* and *BCAR3* by PCR. Then, the potential miR-199-3pbinding sites were inserted into the pmirGLO (Promega) vector to construct luciferase reporter vector. HEK293T cells were transfected withmiR-199a/b-3p mimics or Mir-negative controls (Gene pharma). At the same time, Mut or WT reporter vector was co-transfected into HEK293T cells. Cells were harvested and lysed with extraction buffer48 h after transfection. The results were assayed by Dual-Glo Luciferase Assay System (Promega) according to the manufacturer’s instructions. The nucleotide sequences for *PAK4* 3′UTR were: 50-GACCC TACTA CTGAA CTCCA GT-3′, and the mutagenesis sequences for *PAK4* 30UTR-mut were: 5′-GACCC TACCGTGTAA CTCCA GT-3′. The nucleotide sequences for *BCAR3* 3′UTR were: 5′-GACCC TACTA CTGAA CTCCA GT-3′, and the mutagenesis sequences for *BCAR3* 3′UTR-mut were: 5′-GACCC TACCGTGTAA CTCCA GT-3′.

### Scratch wound assay

Cells were plated in 6-well plates and incubated overnight. The scratches were made using a sterilized 10 μl pipette tip. Then, culture medium was removed to eliminate dislodged cells. The scratch wound was observed at 0 and 24 h. All images were collected under the microscope. All experiments were repeated at least three times.

### Migration and invasion assays

Migration and invasion assays were performed in Transwell chambers (8 μm pore size, 24-well plate; Corning). 600 μl volume of 10% FBS-containing medium was added to the lower chamber and 1 × 10^4^ cells were diluted in a 200 μl volume of serum-free medium were added to the upper chamber. For invasion assay, the upper chambers were coated with diluted Matrigel (BD Biosciences). After incubation for 48 h at 37 °C with 5% CO_2_, cells on the upper surface of the membranes were removed using a cotton swab. The remaining cells were fixed with 4% formaldehyde and stained with 1% crystal violet. The cells were counted and photographed under the inverted microscope in three fields of the view.

### Western blotting

20 μg of total proteins were loaded onto 8–10% polyacrylamide gels and transferred onto polyvinylidene difluoride (PVDF) membranes. The membranes were subsequently blocked with 5% non-fat milk in TBST for 1 h at room temperature. The membranes were incubated with primary antibodies including *PAK4* (Cell Signaling Technology, dilution 1:1000) and *BCAR3* (Bethyl Laboratories, dilution 1:1000) at 4 °C overnight. GAPDH (Santa Cruz Biotechnology, dilution 1:1000) was used as the loading control. After washing 3 times, the membranes were incubated with anti-rabbit or anti-mouse IgG HRP-linked (Cell Signaling Technology, dilution 1:3000) secondary antibody at room temperature for 1 h and visualized by enhanced chemiluminescence detection kit (Thermo Fisher).

### Statistical analysis

All statistical analysis was performed using SPSS 22.0 software. Data were presented as the mean ± SD and differences between groups were analyzed using Student’s *t* test. *P* < 0.05 was considered statistically significant.

## Results

### Silence of *PAK4* inhibited proliferation and colony formation in CRC

**Fig. 1:**
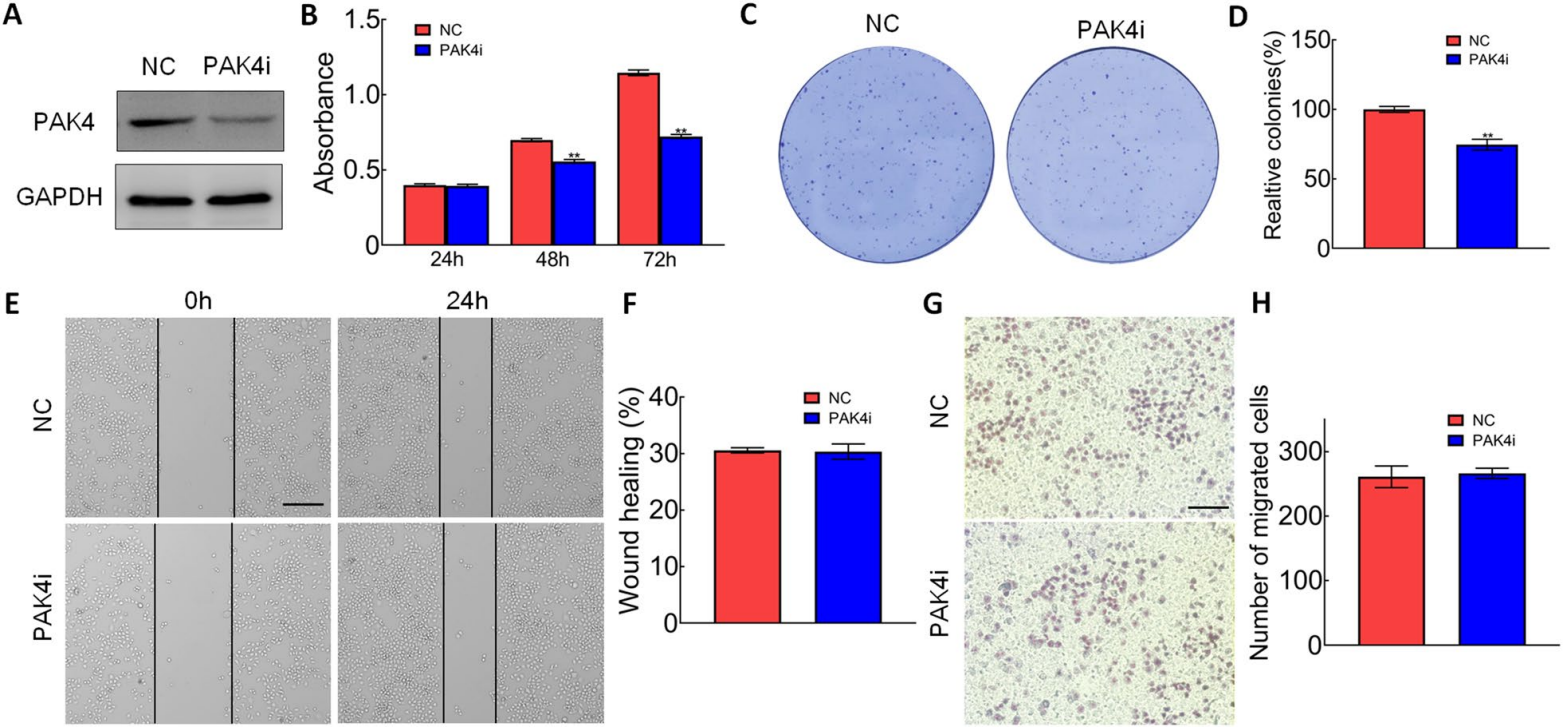
Silence of PAK4 inhibited proliferation and colony formation in CRC. **A** Western blot was used to measure the protein levels of the PAK4 and GAPDH protein in SW1116 cells transfected with PAK4 siRNA expression plasmid. GAPDH was used as the loading control. **B** MTT assay was performed on SW1116 cells transfected with PAK4 siRNA expression plasmid; **p < 0.01. **C**, **D** Colony formation assay was performed on SW1116 cells transfected with PAK4 siRNA expression plasmid; **p < 0.01. **E**, **F** Cell migration was analyzed using scratch wound assay. (bar, 200 μm). **G**, **H** Cell invasion was analyzed using transwell assay. (bar, 100 μm)

To investigate the role of *PAK4* in CRC cells, we silenced *PAK4* expression by transfecting *PAK4* siRNA expression vectors in SW1116 cells. The results from western blot showed that *PAK4* was significantly decreased in the cells transfected with *PAK4* siRNA vectors (Fig. 1A). MTT and colony formation assay indicated that the knockdown of *PAK4* significantly inhibited the proliferation and decreased the number of colonies in SW1116 cells (Fig. 1B–D). However, scratch wound and transwell assays showed that the knockdown of *PAK4* had no significant effect on migration and invasion of SW1116 cells (Fig. 1E–H). These results indicated the role of *PAK4* in colorectal cell proliferation and colony formation.

### Silence of *BCAR3* inhibited cell migration and invasion in CRC

**Fig. 2:**
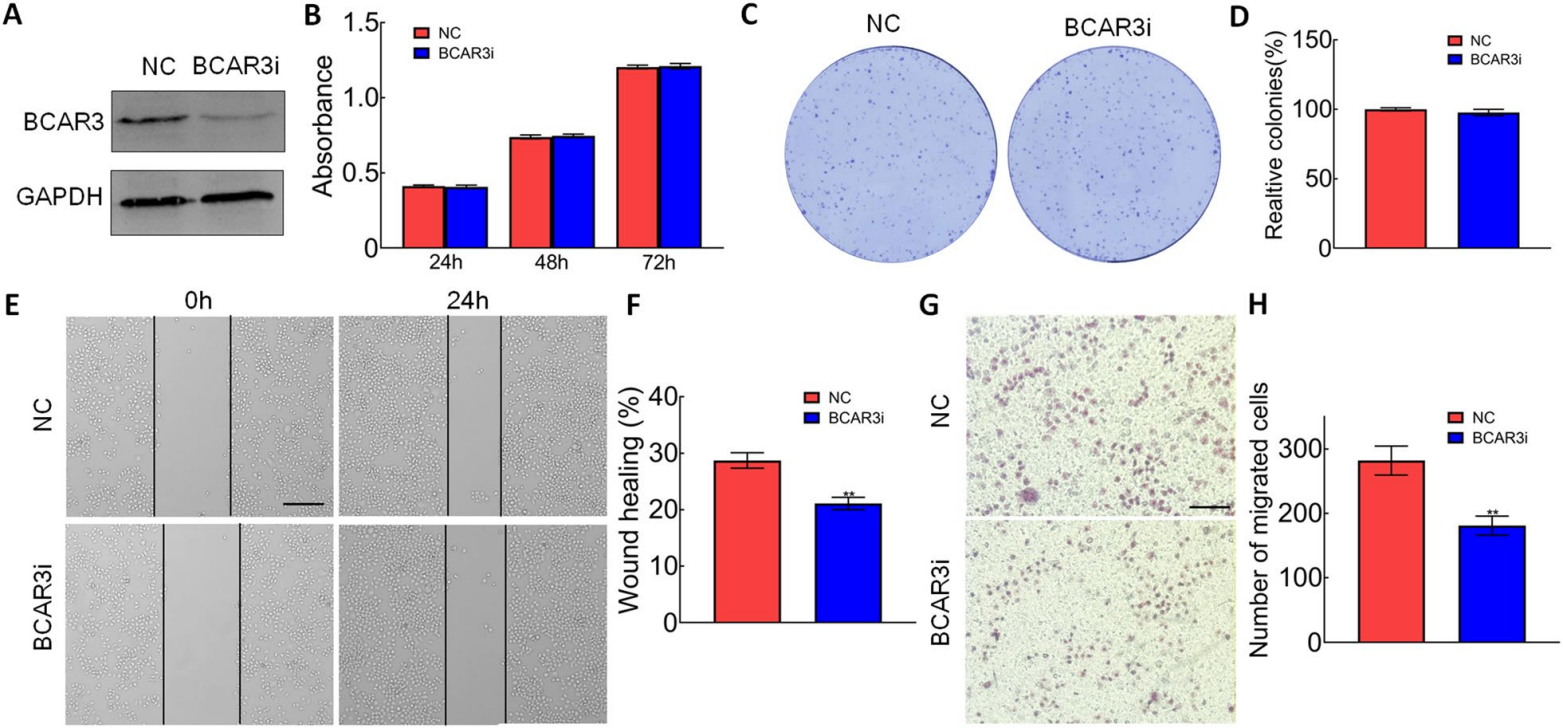
Silence of BCAR3inhibited cell migration and invasion in CRC. **A** Western blot was used to measure the protein levels of the BCAR3 and GAPDH protein in SW1116 cells transfected with BCAR3 siRNA expression plasmid. GAPDH was used as the loading control. **B** MTT assay was performed on SW1116 cells transfected with BCAR3 siRNA expression plasmid. **C**, **D** Colony formation assay was performed on SW1116 cells transfected with BCAR3 siRNA expression plasmid. **E**, **F** Cell migration was analyzed using scratch wound assay (bar, 200 μm); **p < 0.01. (G, H) Cell invasion was analyzed using transwell assay (bar, 100 μm); **p < 0.01

To investigate the role of *BCAR3* in CRC cells, we silenced *BCAR3* expression by transfecting *BCAR3* siRNA expression vectors in SW1116 cells. The results from western blot showed that *BCAR3* was significantly decreased in the cells transfected with *BCAR3* siRNA vectors (Fig. 2A). MTT and colony formation assay indicated that the knockdown of *BCAR3*had no significant effect on proliferation and colony formation in SW1116 cells (Fig. 2B–D). Scratch wound and transwell assays showed that the knockdown of *BCAR3* significantly inhibited cell migration and invasion of SW1116 cells (Fig. 2E–H). These results indicated the role of *BCAR3* in colorectal cell migration and invasion.

### miR-199a/b-3p was the target miRNA of *PAK4* and *BCAR3*

**Fig. 3:**
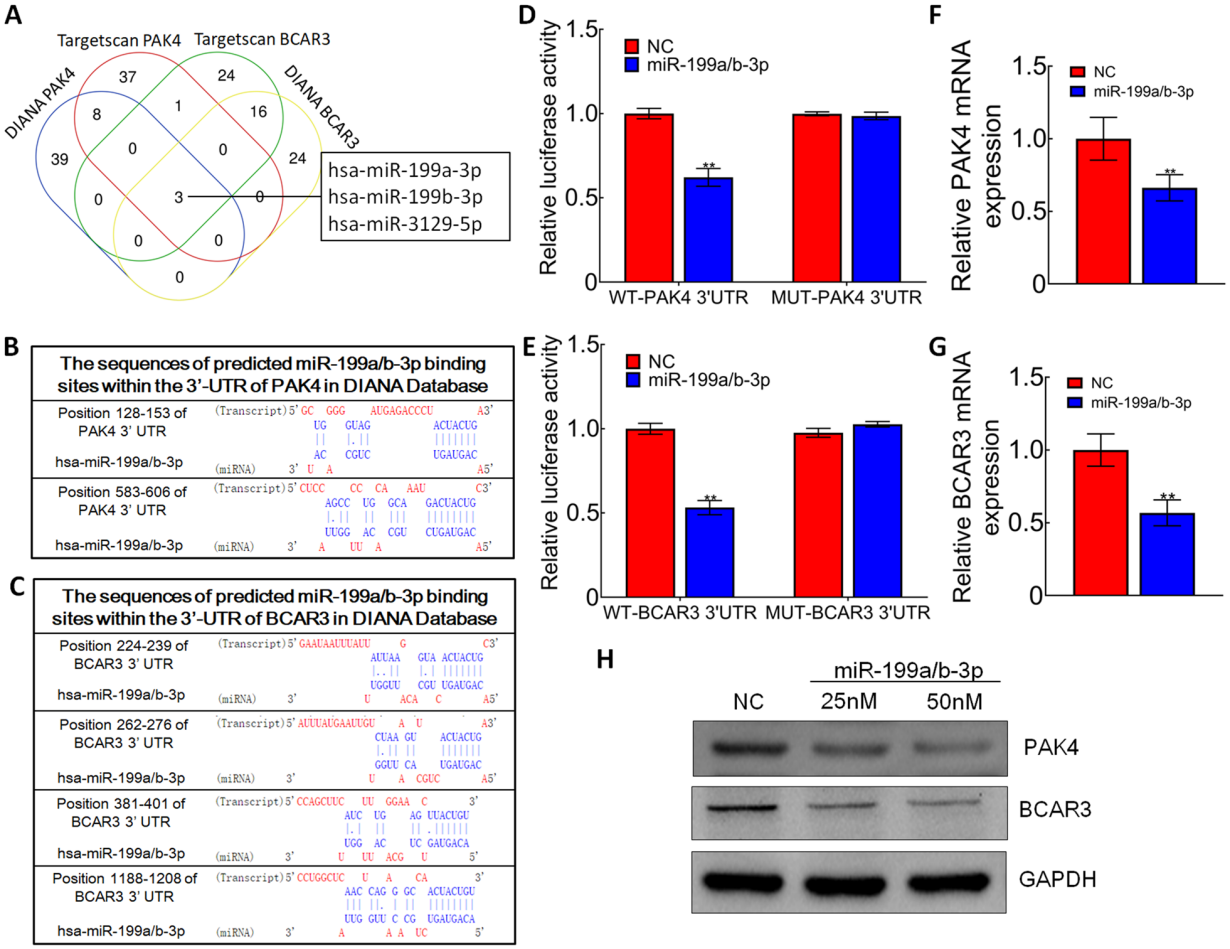
miR‑199a/b-3p was the target of PAK4 and BCAR3. **A** TargetScan and DIANA database were used to predict the target miRNAs of PAK4 and BCAR3. **B** Sequences of predicted miR-199a/b-3p binding sites within PAK4 3’-UTR in DIANA database. **C** Sequences of predicted miR-199a/b-3p binding sites within BCAR3 3’-UTR in DIANA database. **D**, **E** Effect of miR-199a/b-3p on luciferase intensity controlled by the wild type (WT) or mutant (MUT) 3’UTR of PAK4/BCAR3 was determined using the luciferase assay; **p < 0.01. **F**, **G** mRNA levels of PAK4/BCAR3 were detected using quantitative RT-PCR in SW1116 cells transfected with miR-199a/b-3p mimics; **p < 0.01. **H** Protein levels of PAK4 and BCAR3 were detected using western blot in SW1116 cells transfected with miR-199a/b-3p mimics

Given that *PAK4* and *BCAR3* had different roles on CRC cells and the diversity of miRNA targets, we screened out potential target miRNAs of these two genes by bioinformatics analysis. According to the results from TargetScan and DIANA database, we chose miR-199a/b-3p as the target miRNA (Fig. 3A). There are two potential target sites for miR-199a/b-3p at position 128–153 and 583–606 of *PAK4* mRNA 3’UTR (Fig. 3B). There are four potential target sites for miR-199a/b-3p at position 224–239, 262–276,381–401, and 1188–1208 of *BCAR3* mRNA 3’UTR (Fig. 3C). Luciferase reporter assay was conducted to examine the hypothesis that miR-199a/b-3p targeted the 3'-UTR of *PAK4* and *BCAR3*. The results indicated that miR-199a/b-3p significantly decreased the luciferase activity of the reporter with wild type 3’-UTR of *PAK4* and *BCAR3* (Fig. 3D, E). qRT-PCR and western blot analyses verified that miR-199a/b-3p decreased *PAK4* and *BCAR3* expression at mRNA and protein levels in SW1116 cells (Fig. 3F–H). Results showed that miR-199a/b-3p could directly target the 3′-UTR of *PAK4* and *BCAR3*.

### miR-199a/b-3p inhibited cell proliferation and colony formation in CRC

**Fig. 4:**
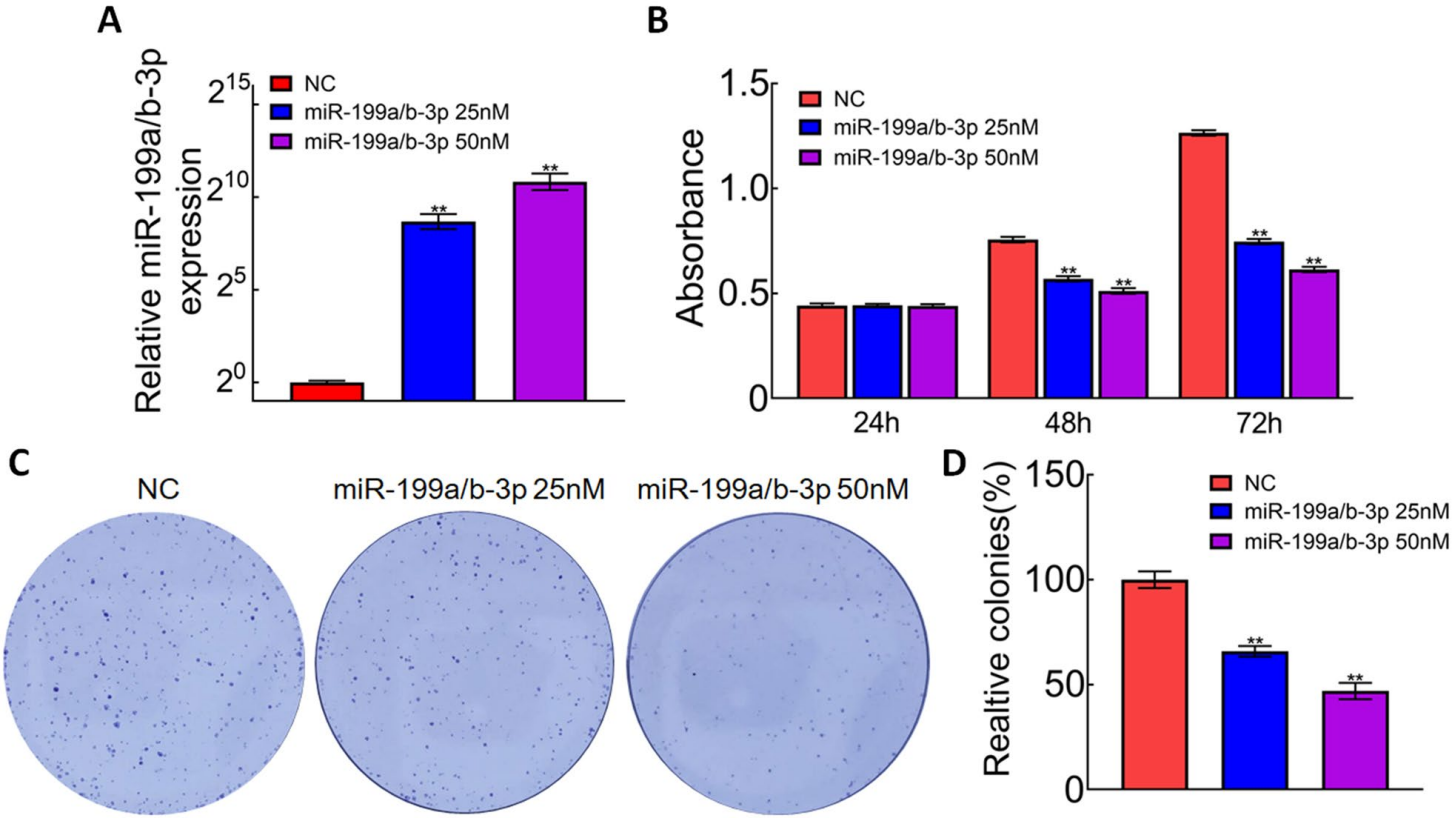
miR-199a/b-3p inhibited cell proliferation and colony formation in CRC. **A** Levels of miR-199a/b-3p were detected using quantitative RT-PCR in SW1116 cells transfected with miR-199a/b-3p mimics; **p < 0.01. **B** MTT assay was performed on SW1116 cells transfected with miR-199a/b-3p mimics; **p < 0.01. **C**, **D** Colony formation assay was performed on SW1116 cells transfected with miR-199a/b-3p mimics; **p < 0.01

To further explore the role of miR-199a/b-3p in colorectal cells, we transfected the miR-199a/b-3p mimics or negative controls into SW1116 cells. qRT-PCR was used to detect the relative expression of miR-199a/b-3p. The level of miR-199a/b-3p was significantly increased in mimics transfected cells (Fig. 4A). MTT and colony formation assays indicated that miR-199a/b-3p mimics inhibited the proliferation and decreased the number of colonies in SW1116 cells (Fig. 4B–D).

### miR-199a/b-3p inhibited cell migration and invasion in CRC

**Fig. 5:**
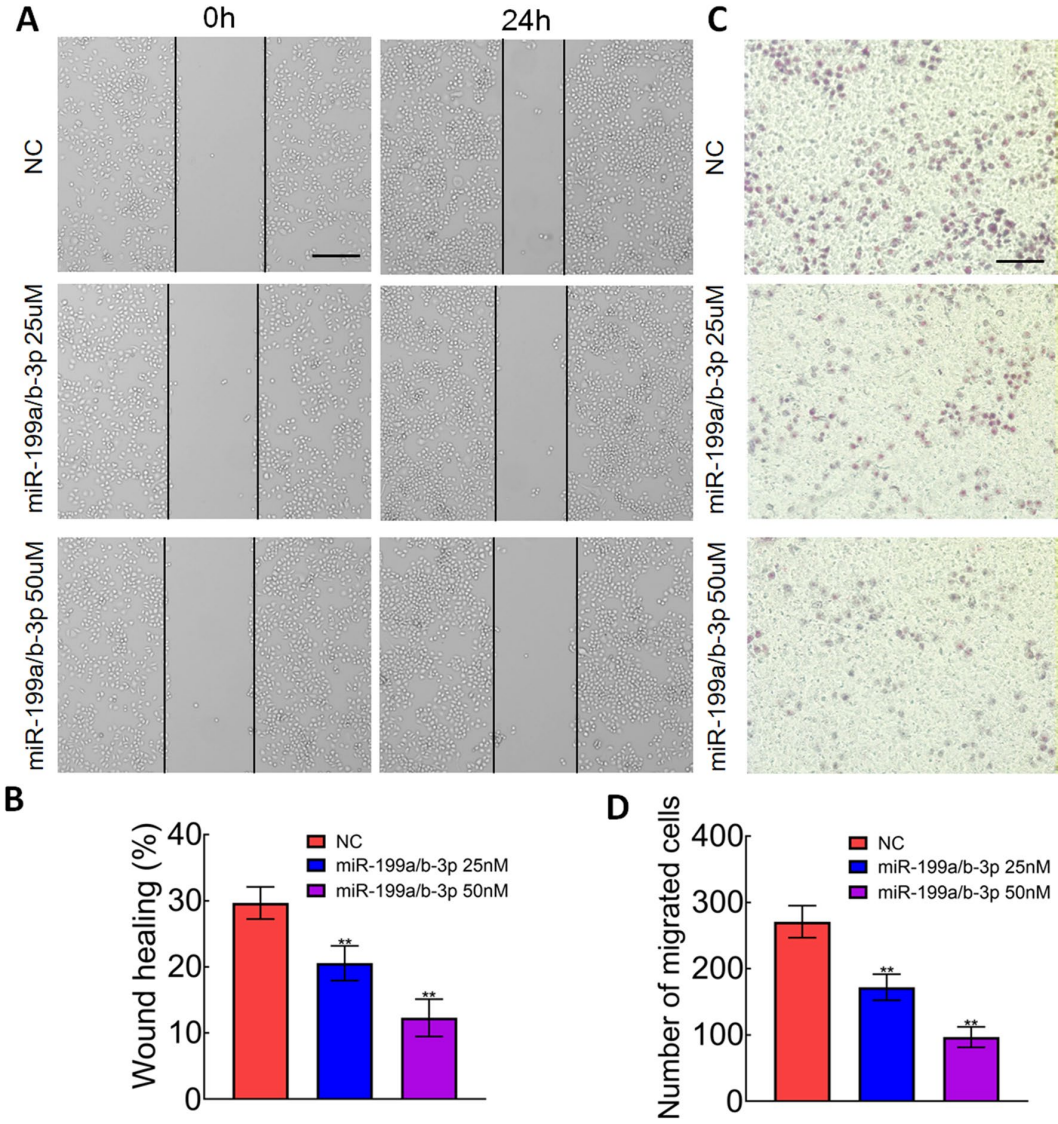
miR-199a/b-3p inhibited cell migration and invasion in CRC. **A**, **B** SW1116 cells were transfected with miR-199a/b-3p mimics. Cell migration was analyzed using scratch wound assay. (bar, 200 μm). **C**, **D** SW1116 cells were transfected with miR-199a/b-3p mimics. Cell invasion was analyzed using transwell assay. (bar, 100 μm)

We then determined the effect of miR-199a/b-3p on migration and invasion. Scratch wound assay results showed that themiR-199a/b-3p mimics significantly inhibited the migratory ability of SW1116 cells (Fig. 5A, B). Transwell assay results showed that miR-199a/b-3p mimics also significantly inhibited the invasive ability of SW1116 cells (Fig. 5C, D). These results indicated that miR-199a/b-3p could suppress cell migration and invasion in CRC cells.

### The effects of miR-199a/b-3p on cell proliferation, migration, and invasion could be partially reversed by *PAK4* and *BCAR3* overexpression

**Fig. 6:**
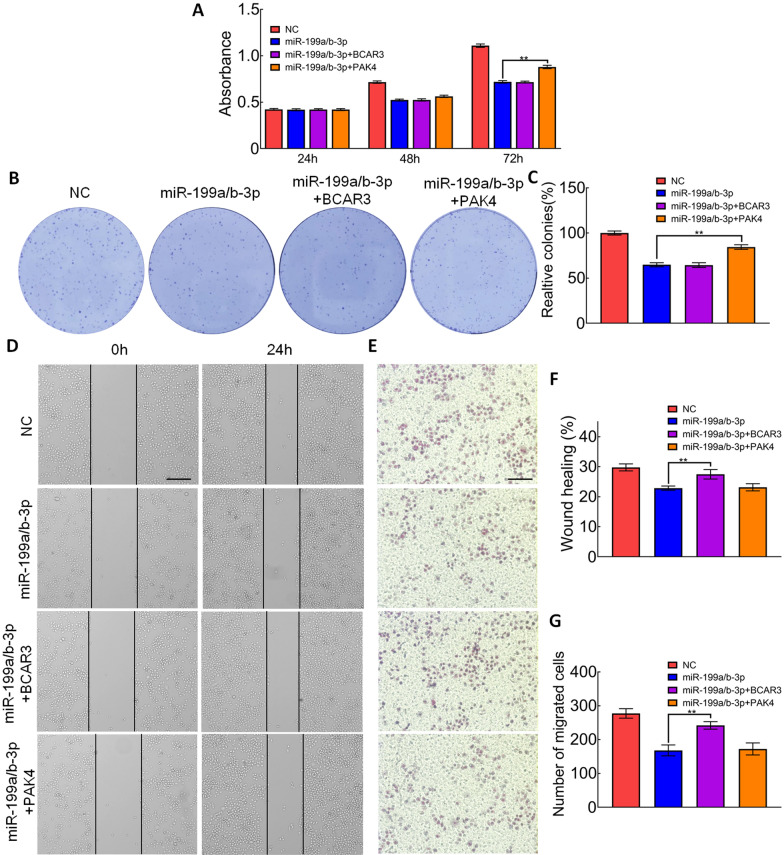
Effects of miR-199a/b-3p could be partially reversed by PAK4 and BCAR3 overexpression. **A** MTT assay was performed on SW1116 cells transfected with miR-199a/b-3p mimics and/or PAK4/BCAR3 overexpression plasmid; **p < 0.01. **B**, **C** Colony formation assay was performed on SW1116 cells transfected with miR-199a/b-3p mimics and/or PAK4/BCAR3 overexpression plasmid; **p < 0.01. **D**, **F** Cell migration was analyzed using scratch wound assay. (bar, 200 μm);**p < 0.01. **E**, **G** Cell invasion was analyzed using transwell assay. (bar, 100 μm);**p < 0.01

Since the inhibitory effects of miR-199a/b-3p included the effects of silencing *PAK4* and *BCAR3*, we examined that whether the function could be reversed by overexpressing *PAK4* or *BCAR3*. We transfected negative controls, miR-199a/b-3p mimics, miR-199a/b-3p+*BCAR3*, or miR-199a/b-3p+*PAK4* in SW1116 cells. MTT and colony formation assays showed that the restoration of *PAK4* expression reversed the effect of miR-199a/b-3p on cell proliferation and colony formation (Fig. 6A–C). The results from scratch wound and transwell assays showed that ectopic *BCAR3* expression reversed the inhibitory effect of miR-199a/b-3p on cell migration and invasion (Fig. 6D–G). The above results indicated that miR-199a/b-3p inhibited viability and mobility of CRC cells by targeting *PAK4* and *BCAR3,* respectively.

## Discussion

CRC is a complex disease and one of the main causes of death from cancer worldwide [19, 20]. The morbidity of CRC is increasing every year. Meanwhile the global incidence of CRC is expected to increase to 2.5 million by 2035 [21]. Although the life expectancy for CRC patients has increased as a result of advances in screening and treatment, the prognosis of these patients remains poor [22]. Therefore, it is of great significance to understand the underlying mechanism of its occurrence and development and to find out the approaches to overcome the poor prognosis.

*PAK4* has been reported to be involved in the regulation of cell cycle regulatory proteins p21, p16, and CDK6 [23]. Studies showed that the down-regulation of *PAK4* had inhibitory effects on CRC cells [10, 11].Consistent with these previous reports, our study demonstrated that the knockdown of *PAK4* significantly inhibited the proliferation and colony formation of SW1116 cells. However, there was no significant effect on migration and invasion.

Recently, reports have shown that the overexpression of *BCAR3* could regulate cytoskeletal rearrangement and improved the cell migration and invasion in endometriosis [13, 14]. However, there no relevant studies conducted on the role of *BCAR3* in CRC. Therefore, we knocked down the expression of *BCAR3* to explore the role of *BCAR3* in CRC. Results showed that the knockdown of *BCAR3* significantly inhibited cell migration and invasion of SW1116 cells, but there was no significant effect on proliferation and colony formation.

MiRNAs are endogenous non-coding small RNAs, which play important roles in plants and animals [24]. They inhibit the gene translation or degrading the target mRNA by binding to the 3ʹ-untranslated region (3ʹ-UTR) of target genes [25]. A single miRNA can target numerous mRNA and regulate the expression of multiple genes, which involved in cancer progression in multiple ways. Much more studies have shown that miRNAs play essential roles in promoting or inhibiting the proliferation, invasion, apoptosis and drug resistance of tumor cells by regulating some key genes [26–28]. Some studies revealed that miRNAs are involved in progression of CRC [29, 30].

Considering the different roles of *PAK4* and *BCAR3* on CRC cells and the multiple targets of miRNA, we decided to predict the potential target miRNAs of *PAK4* and *BCAR3*. From the results in TargetScan and DIANA database, we surprisingly found miR-199a/b-3p could be the target miRNA of both genes. Our luciferase reporter assay results showed that *PAK4* and *BCAR3* were potential targets of miR-199a/b-3p in SW1116 cells. Moreover, qRT-PCR and western blot analyses verified that miR-199a/b-3p decreased *PAK4* and *BCAR3* expression at mRNA and protein levels in SW1116 cells, which meant that miR-199a/b-3p could directly target the 3'-UTR of *PAK4* and *BCAR3*.

As for miR-199a/b-3p, there are several studies of its suppressive role in tumor progression. MiR-199a/b-3p could suppress hepatocellular carcinoma through inhibiting *PAK4/Raf/MEK/ERK* pathway [31]. In this study, our results indicated that miR-199a/b-3p significantly inhibited the proliferation, colony formation, migration, and invasion of SW1116 cells.

We then performed rescue experiments to explore whether *PAK4* and *BCAR3* were downstream functional regulators involved in miR-199a/b-3p regulation in SW1116 cells. Overexpression of *PAK4* significantly alleviated the effects of miR-199a/b-3p in SW1116 cells on cell proliferation and colony formation. Meanwhile, the overexpression of *BCAR3* significantly reversed the effects of miR-199a/b-3p in SW1116 cells on cell migration and invasion. The above results indicated that miR-199a/b-3p inhibited the proliferation, colony formation of SW116 cells through targeting *PAK4*. Meanwhile, the inhibitory effects on migration and invasion were regulated by targeting *BCAR3*.

In summary, our research demonstrated that miR-199a/b-3p inhibited viability and mobility of CRC cells by regulating *PAK4* and *BCAR3,* respectively. This study proposed a new miRNA for filtrating multi-targeted neoplastic suppressant to improve the prognosis of CRC in patients.

## Data Availability

All data supporting the conclusions of this article are included within the article.

## Abbreviations

CRC: Colorectal cancer
*PAK4*: P21 activated kinase 4
*BCAR3*: The breast cancer anti-estrogen resistance 3
SW116: Human colorectal cancer cells
MTT: 3-(4,5-Dimethylthiazol-2-yl)-2,5-diphenyltetrazolium bromide
DMSO: Dimethyl sulfoxide
PBS: Phosphate-buffered saline
PVDF: Polyvinylidene fluoride
